# Egg Quality and Nutritional Profile of Three Sicilian Autochthonous Chicken Breeds: Siciliana, Cornuta di Caltanissetta, and Valplatani

**DOI:** 10.3390/foods14152571

**Published:** 2025-07-22

**Authors:** Vittorio Lo Presti, Francesca Accetta, Maria Elena Furfaro, Antonino Nazareno Virga, Ambra Rita Di Rosa

**Affiliations:** 1 Dipartimento di Scienze Veterinarie, University of Messina, Viale Palatucci, 98168 Messina, Italy; vittorio.lopresti@unime.it (V.L.P.); francesca.accetta@studenti.unime.it (F.A.); 2 Meat and Agribusiness Chain Research Consortium, Viale Palatucci, 98168 Messina, Italy; mariaelena.furfaro@corfilcarni.it (M.E.F.); antoninovirga58@gmail.com (A.N.V.)

**Keywords:** autochthonous poultry, biodiversity conservation, eggs fatty acids, eggs nutritional indices

## Abstract

The conservation of poultry biodiversity is a growing global priority, yet it necessarily relies on the scientific valorization of specific local breeds. This study aimed to characterize the lipid composition and cholesterol content of eggs from three native Sicilian chicken breeds (Cornuta, Valplatani, and Siciliana) reared under semi-extensive conditions, in order to evaluate their nutritional potential and support biodiversity preservation strategies. A total of 170 eggs from 11 farms were analyzed. Fatty acid composition and nutritional indices (atherogenic index, thrombogenic index, n-6/n-3 ratio, HH index) were determined according to ISO and AOAC standards. Results showed that Cornuta eggs exhibited the most favorable lipid profile, with the lowest saturated fatty acid (SFA) content (38.55%), the lowest n-6/n-3 ratio (7.35), and the best values for AI (0.52), TI (1.22), and HH (2.02), compared to Valplatani and Siciliana. Conversely, the lowest cholesterol content was found in Siciliana eggs (1463.58 mg/kg), significantly lower than Cornuta (1789 mg/kg; *p* < 0.05). Although no commercial hybrids were included, the literature data were used for contextual comparison. These findings suggest that native breeds may produce eggs with functional nutritional properties, supporting both healthier food choices and local genetic conservation. Moreover, this study provides a replicable framework for the nutritional valorization of underutilized poultry breeds, reinforcing the role of biodiversity in sustainable food systems.

## 1. Introduction

Conserving native poultry breeds is an important aspect of sustainable livestock strategies, particularly in marginal and biodiversity-rich regions. Local poultry breeds, shaped over centuries by natural adaptation to specific environmental and socio-cultural contexts, represent a unique reservoir of biodiversity. However, they currently face increasing threats from the expansion of commercial hybrids and ongoing genetic erosion [1,2].

In Sicily, a region with deep-rooted rural traditions, several historical poultry populations have been officially recognized as Italian native breeds. The Siciliana breed has long been included in the national registry, whereas the Cornuta di Caltanissetta (Ministry of Agriculture, Food Sovereignty and Forestry, Decree No. 0140687 of 27 March 2025) and Valplatani (MASAF Decree No. 184335 of 23 April 2024) breeds have only recently received official recognition. The official recognition of native poultry breeds could open opportunities for niche marketing, access to conservation funding, and inclusion in genetic improvement programs [3]. Although previous studies have assessed productive and qualitative traits of several Italian native chicken breeds [4,5,6], no study to date has performed a comparative nutritional profiling—specifically in terms of fatty acid composition, cholesterol content, and lipid quality indices—of the eggs from the three Sicilian breeds included in this research. This gap is particularly relevant in the context of promoting biodiversity through the valorization of underutilized genetic resources.

This study aims to characterize and compare the egg lipid profile, cholesterol content, and nutritional indices of three Sicilian native chicken breeds reared under semi-extensive conditions, in order to assess their potential as functional food sources and support conservation-oriented valorization.

These breeds exhibit distinct phenotypic and morphological traits that may influence their adaptive capacity and egg quality [7]. The Siciliana, one of the oldest documented breeds in Italy (dating back to the 17th century), is characterized by a rose comb, golden or silver plumage, and a slender, upright body. It is valued for both egg-laying performance and ornamental features. The Cornuta di Caltanissetta, also known as Cornutella or Corna di Bue, originates from the hills and mountains of central Sicily and is notable for its bifid comb, wild-type plumage, and robust musculature. It displays considerable resistance to disease and environmental stress. The Valplatani, named after the Platani River valley in central-western Sicily, is compact in size, reddish in color, and bears a single comb. It is typically reared in small-scale rural systems and is well adapted to marginal territories, with moderate laying capacity.

Genomic studies have confirmed the uniqueness of these populations. In particular, the Cornuta forms a distinct genetic cluster, whereas the Valplatani shows signs of admixture, possibly due to historical introgressions with commercial lines [8,9]. Morphological differences may also affect metabolic efficiency and reproductive traits. For example, the Cornuta’s pronounced musculature may favor lipid deposition and influence yolk fatty acid composition, while the Valplatani’s smaller size and rustic management may contribute to variability in nutrient assimilation. These hypotheses align with previous studies linking genotype, phenotype, and egg composition in native Italian breeds [4,5,6,9].

Sicilian poultry breeds, therefore, play a strategic role not only as genetic heritage to be preserved but also as a sustainable resource for resilient, locally adapted farming systems. Their continued use supports rural development, biodiversity valorization, and the creation of short food supply chains based on origin-certified products [1,2].

Eggs from native breeds are considered foods of high biological value, whose chemical and nutritional composition may reflect both genotype and environmental conditions. Several studies have shown that breed, diet, and farming system significantly influence egg quality traits, particularly lipid composition, fatty acid balance, and mineral profile [4,5,6].

In recent years, growing consumer awareness of nutrition, sustainability, and traceability has driven renewed interest in niche animal products with specific health-oriented characteristics. Eggs are widely recognized as a complete and affordable food, rich in high-quality proteins, bioavailable minerals, and essential lipids. Their nutritional profile is strongly influenced by genetic background and feeding conditions [1,4,6].

Autochthonous poultry breeds, long overlooked by commercial selection, are now increasingly valued for their ability to produce functional animal-derived foods compatible with modern dietary models [2,5]. Although nutritional evaluation alone may not directly determine conservation priorities, it can provide functional data to support valorization initiatives aimed at integrating native breeds into sustainable food chains. Such approaches are increasingly promoted within biodiversity strategies, including those endorsed by FAO and national genetic resource programs.

Within this framework, the present study aimed to assess the chemical composition, fatty acid profile, mineral content, and nutritional indices of eggs from three Sicilian native breeds—Siciliana, Cornuta di Caltanissetta, and Valplatani—highlighting breed-specific differences and identifying relevant traits for conservation and functional food development.

We hypothesized that the eggs of the three Sicilian native chicken breeds, reared under similar semi-extensive conditions, would exhibit distinct lipid and cholesterol profiles reflecting breed-specific nutritional traits. These differences could provide a scientific basis for their nutritional valorization and contribute to biodiversity-oriented food strategies.

## 2. Materials and Methods

### 2.1. Sample Collection and Origin

**Figure 1:**
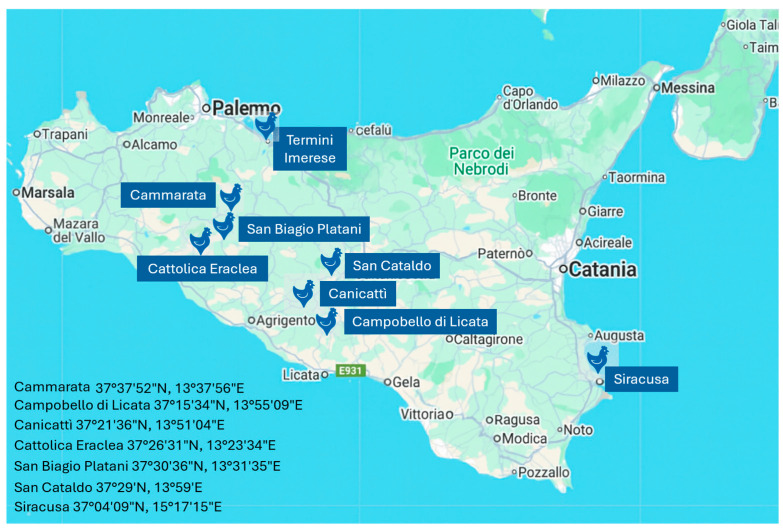
Geographic location of the 11 farms involved in egg sampling across eight municipalities in Sicily. The map was created using Google Maps and reflects the approximate position of each farm.

The eggs were collected from three Sicilian autochthonous chicken breeds—Siciliana, Cornuta di Caltanissetta, and Valplatani—registered in the Italian National Poultry Register (Ministry of Agriculture, Food Sovereignty and Forestry, Decree No. 0140687 of 27 March 2025 and MASAF Decree No. 184335 of 23 April 2024). Sampling was conducted across 11 traditional farms located in 8 municipalities within the provinces of Caltanissetta, Agrigento, Siracusa, and Palermo (Sicily, Italy) (Figure 1). The study included a total of 170 eggs, distributed across three genetic groups: Cornuta (n = 50), Valplatani (n = 59), and Siciliana (n = 61). Hens were reared under semi-extensive conditions with access to outdoor areas and were fed low-input, cereal-based diets composed mainly of locally grown grains (barley, wheat, and maize), without the use of synthetic additives or lipid-enriching supplements.

Sampling was carried out during the winter period (November 2024 to March 2025). All eggs were obtained from hens older than 35 weeks, corresponding to the mid-laying stage, which may affect egg composition. Although no environmental parameters (e.g., temperature, humidity) were directly measured, all farms were located within a relatively small geographical area of Sicily, characterized by mild and homogeneous winter climate conditions. Therefore, climatic variability was not considered a relevant factor influencing the results.

Each of the 170 eggs was individually labeled at the time of collection. Chemical and nutritional analyses were performed on a single-egg basis to preserve individual biological variability. Samples were stored at 4 °C and processed within 48 h of arrival at the laboratory.

Although the study did not include a commercial hybrid control group, comparative interpretation was supported by published data on widely used commercial layers (e.g., Hy-Line, Warren, Isa Brown). Given the aim of valorizing underutilized local genetic resources, a literature-based benchmark was considered appropriate to contextualize the nutritional potential of these native breeds within broader poultry production systems.

### 2.2. Chemical Analysis

Proximate composition, including moisture, crude protein, total fat, and ash contents, was determined using standard gravimetric and chemical procedures. Moisture content was determined according to ISTISAN Reports 1996/34 Met. B [10]; crude protein by the Kjeldahl method (ISO 1871:2009) [11]; total fat content by ISTISAN 1996/34 page 41 Met A, [10]; and ash content by ISTISAN Reports 1996/34 page 77 [10].

Cholesterol content was quantified according to the AOAC Official Method 994.10 [12], which involves direct saponification. After saponification, the cholesterol is extracted and analyzed using gas chromatography with flame ionization detection (GC-FID), according to validated procedures described in previous studies [5].

Mineral profiling was carried out by inductively coupled plasma mass spectrometry (ICP-MS) after microwave-assisted acid digestion. Digestion conditions for the microwave system applied were as follows: up to 180 °C for 20 min and then constant for 30 min; finally, a cooling stage (30 min) was carried out to 22 °C, and samples were diluted to 50 mL with deionized ultrapure water. This solution was filtered with membrane filters (0.45 µm) and performed with an ICP-MS equipped with a concentric Nebulizer, a quartz torch with quartz injector tube, and a cyclonic spray chamber. External calibration curves from standard solutions were used to quantify the amount of sodium (Na), potassium (K), phosphorus (P), calcium (Ca), magnesium (Mg), iron (Fe), zinc (Zn), copper (Cu), selenium (Se), and manganese (Mn) according to UNI EN ISO 17294-2:2016 [13].

Fatty Acid Profile: Total lipids were extracted from the yolks using a chloroform–methanol mixture (2:1, *v*/*v*). The lipids were then transmethylated to fatty acid methyl esters (FAMEs) with a methanolic solution of potassium hydroxide at room temperature.

Approximately 0.1 g of the oil sample was accurately weighed into a 5 mL screw-top test tube. Two milliliters of heptane and 0.2 mL of methanolic potassium hydroxide solution were then added. The mixture was allowed to stratify until the upper layer became clear. The resulting supernatant was analyzed using a Shimadzu 2010 PRO gas chromatograph (GC). The GC was equipped with a split injector, a flame ionization detector (FID), and a CP-Sil 88 fused silica capillary column (100 m × 0.25 mm I.D., 0.25 μm film thickness). Analysis followed ISO 12966-2:2017 [14] and Di Rosa et al. [5].

Column temperature ranged from 100 °C to 240 °C with a heating rate of 4 °C/min and a final isotherm at this temperature for 20 min. Both the injector and detector temperatures were maintained at 250 °C. A 1 μL sample volume was injected. Helium was used as the carrier gas at a flow rate of 1 mL/min, with a split ratio of 1:50.

Fatty acids were identified by comparing their retention times with reference compounds (Supelco 37-component FAME mix). Chromatographic peak areas were acquired using the LabSolution instrument control and data analysis software (Shimadzu). FAME quantification was based on internal calibration using tricosanoic acid (C23:0) as the internal standard.

### 2.3. Nutritional Indices

In addition to the quantitative assessment of individual fatty acids, the use of composite indices enables a more comprehensive evaluation of lipid quality from a health perspective. The atherogenic index (AI) and thrombogenic index (TI), introduced by Ulbricht and Southgate, provide insights into the potential of dietary fats to promote atherosclerosis and thrombosis by quantifying the relative proportions of pro- and anti-atherogenic fatty acids [15]. The hypocholesterolaemic/hypercholesterolaemic ratio (HH), proposed by Santos-Silva et al. [16], reflects the potential influence of different fatty acids on plasma cholesterol metabolism [15]. These indices are increasingly used in nutritional research to assess the functional properties of animal-derived foods, such as eggs, and help identify products with potential health-promoting effects.

The following indices were calculated to evaluate the nutritional quality of the yolk lipid fraction:

Atherogenic index (AI), (1) and thrombogenic index (TI), (2) as per Ulbricht and Southgate [14]:

(1) AI = (C12:0 + 4 × C14:0 + C16:0)/(∑MUFA + ∑PUFA)

(2) TI = (C14:0 + C16:0 + C18:0)/[0.5 × ∑MUFA + 0.5 × ∑n-6 + 3 × ∑n-3 + (n-3/n-6)]

Hypocholesterolaemic/hypercholesterolaemic Ratio (HH), (3) according to Santos-Silva et al. [15]:

(3) HH = (∑C18:1 + ∑PUFA)/(C14:0 + C16:0)

Omega-6/omega-3 ratio (n-6/n-3) (4):

(4) n-6/n-3 = ∑(n-6 fatty acids)/∑(n-3 fatty acids)

Peroxidation index (PI), (5) according to Arakawa and Sagai [17]:

(5) PI = (0.025 × %monoenes) + (1 × %dienes) + (2 × %trienes) + (4 × %tetraenes) + (6 × %pentaenes) + (8 × %hexaenes)

### 2.4. Statistical Analysis

The study followed a completely randomized design, with individual eggs considered as experimental units. Levene’s test was used to assess the homogeneity of variances, while the Shapiro–Wilk test evaluated the normality of residuals. Although some variables exhibited mild deviations from normality, the assumption of homoscedasticity was met, and group sizes were sufficiently balanced. Given the robustness of one-way ANOVA to moderate violations of normality—especially under equal group sizes—the fixed effect of genetic type on each measured parameter was evaluated using one-way ANOVA. A Kruskal–Wallis non-parametric test was also performed in parallel to confirm the consistency of the results. Post hoc comparisons were carried out using Tukey’s HSD test.

In addition, a principal component analysis (PCA) was performed to explore multivariate relationships among the measured variables.

All statistical analyses were conducted using XLStat software, Vers. 2021.2.2 (Addinsoft, Paris, France, 2025) [18].

### 2.5. Language Editing Support

A professional version of ChatGPT (GPT-4, 2025) was used to support the preparation of this manuscript. The tool was employed exclusively to assist with language refinement, terminology consistency, and formatting in accordance with journal guidelines. All scientific content, data interpretation, and conclusions were entirely conceived, validated, and approved by the authors.

## 3. Results and Discussion

The chemical composition of the eggs showed significant variation among the three Sicilian native breeds (Table 1). Cornuta eggs exhibited the highest average total lipid content in the eggs (10.95%), significantly exceeding the values observed in Siciliana (9.08%) and Valplatani (10.33%) eggs (*p* < 0.05). Cholesterol concentration followed a similar trend, with Cornuta yolks showing the highest values (1789 mg/100 g yolk), which may suggest a higher lipogenic activity, although no metabolic or gene expression data were collected to confirm this hypothesis [19]. No significant differences were found for protein and ash contents, whereas Siciliana eggs displayed a significantly higher moisture content compared to the other two breeds (*p* < 0.05).

In addition to basic chemical composition, the mineral profile of the eggs was assessed to evaluate potential breed-related differences in micronutrient deposition and physiological relevance.

Mineral concentrations were generally consistent across the three groups (Table 2). However, sodium levels were significantly lower in Cornuta eggs (*p* < 0.05), which may reflect breed-related differences in sodium deposition. No physiological measurements (e.g., intestinal absorption, renal excretion, or ion transport) were conducted in this study, and this interpretation should be considered speculative.

Although overall differences were limited, the significantly lower sodium levels observed in Cornuta eggs may have physiological relevance. Sodium plays a central role not only in maintaining osmotic balance but also in the formation of eggshells, where it is actively transported through the shell gland epithelium and contributes to carbonate equilibrium in the uterine fluid [20]. Breed-specific differences in renal reabsorption efficiency or ion transporter expression could underlie the variation observed.

Additionally, trace minerals such as zinc, manganese, and copper—although not significantly different across groups—are known cofactors of key enzymes involved in shell mineralization and membrane stability, such as carbonic anhydrase and alkaline phosphatase [21]. These elements may also affect the bioaccessibility of other nutrients, with potential implications for both egg quality and nutritional value. Recent studies have reported differential mineral deposition and shell composition between native and commercial genotypes, linked to the expression of calcium and sodium transporter genes [22]. Although our study did not assess shell characteristics, transporter activity, or mineral bioavailability directly, these findings suggest directions for future research focused on mineral metabolism and nutrient utilization in autochthonous breeds.

**Figure 2:**
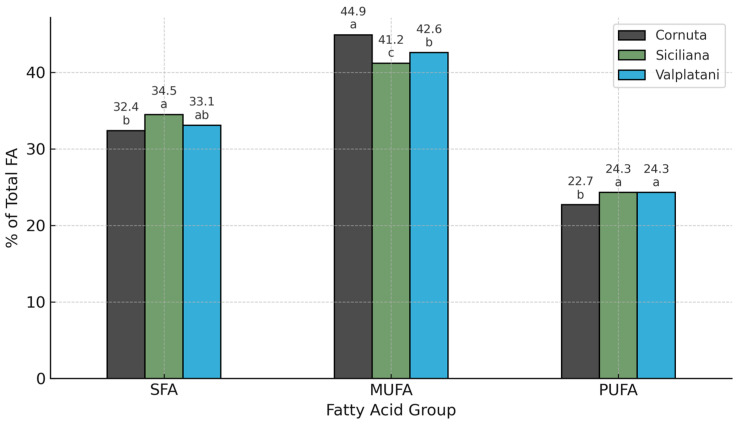
Fatty acid profile (% of total fatty acids) in eggs from Cornuta, Siciliana, and Valplatani breeds. Values are expressed as means. Different letters (a, b, c) above bars indicate statistically significant differences between breeds within each fatty acid group (*p* < 0.05).

To better understand the lipid quality of the eggs, the fatty acid composition of the yolks was analyzed, with particular attention to health-related indices such as MUFA, PUFA, and the omega-6/omega-3 ratio (Figure 2).

The fatty acid profile revealed pronounced breed-related differences (Table 3). Cornuta eggs had a significantly lower proportion of saturated fatty acids (SFA) and a higher content of monounsaturated fatty acids (MUFA), especially oleic acid (C18:1n9), in comparison to Siciliana and Valplatani (*p* < 0.05). The oleic acid content in Cornuta reached 50.74%, compared to 39.92% in Siciliana and 43.36% in Valplatani. Conversely, palmitic acid (C16:0) was significantly lower in Cornuta, supporting a more favorable lipid profile for human health.

No significant differences were observed for total polyunsaturated fatty acids (PUFA); however, Cornuta eggs had significantly higher levels of omega-3 fatty acids—particularly alpha-linolenic acid (C18:3n-3) and eicosapentaenoic acid (C20:5n-3)—although their absolute values remained below 1%. Despite the low quantities, such differences may still contribute to overall dietary omega-3 intake, especially in the context of regular consumption and cumulative dietary sources. This resulted in a more desirable omega-6/omega-3 ratio in Cornuta eggs (7.35), approaching values recommended for human nutrition.

To complement the fatty acid data, composite nutritional indices were calculated to provide an integrated assessment of lipid quality and potential cardiovascular benefits.

**Figure 3:**
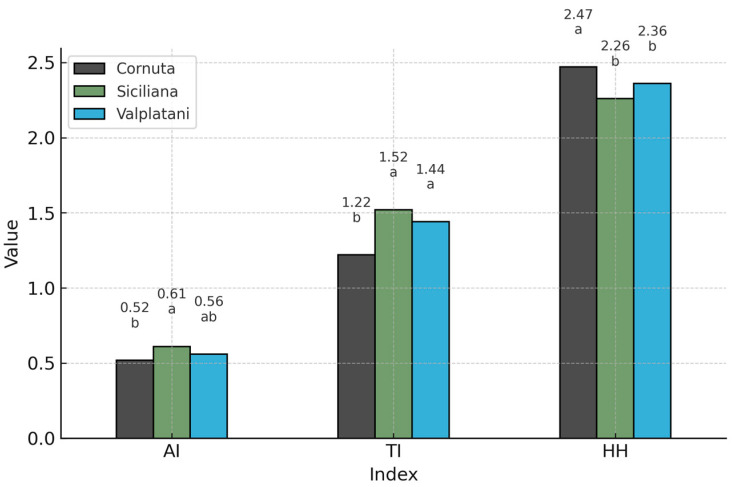
Health-related nutritional indices in eggs from Cornuta, Siciliana, and Valplatani breeds. Values represent mean index values: n-6/n-3 ratio, atherogenic index (AI), thrombogenic index (TI), and hypocholesterolemic/hypercholesterolemic ratio (HH). Different letters (a, b) indicate statistically significant differences between breeds within each index (*p* < 0.05).

Nutritional indices further confirmed the superior profile of Cornuta eggs (Table 4). The atherogenic index (AI) and thrombogenic index (TI) were significantly lower (AI: 0.52; TI: 1.22), while the hypocholesterolaemic/hypercholesterolaemic ratio (HH) was significantly higher (2.02) compared to the other two breeds (*p* < 0.05) (Figure 3). The peroxidation index (PI) did not differ significantly among breeds, indicating similar oxidative stability of yolk lipids.

Eggs from the three Sicilian local chicken breeds—Cornuta, Siciliana, and Valplatani—exhibited distinctive lipid and nutritional profiles, reflecting interactions between genetic heritage and environmental conditions. Cornuta eggs exhibited the highest levels of total lipids and cholesterol. While these values may raise nutritional concerns, they should be interpreted in the context of the overall lipid profile. Cornuta eggs were characterized by a high proportion of MUFA and PUFA, especially alpha-linolenic acid (C18:3n-3), a precursor of long-chain omega-3 fatty acids. These compounds are recognized for their cardioprotective, lipid-modulating, and anti-inflammatory properties [23].

The nutritional indices—AI, TI, and HH—further support the favorable lipid quality of Cornuta eggs. Compared with the other breeds, Cornuta eggs exhibited significantly lower AI and TI and higher HH values, suggesting a reduced cardiovascular risk and improved nutritional benefit [15,24]. In our study, all hens received comparable low-input diets based on local cereals, without any intentional lipid supplementation. The analytical feed composition (crude protein 17.50%, crude fat 5.20%) confirms the non-enriched nature of the formulation. While dietary composition is known to affect yolk lipid profiles, the standardization across farms reduces its role as a confounding factor. Therefore, the observed differences in fatty acid composition are more plausibly attributed to breed-specific metabolic traits. This interpretation is supported by recent findings showing that even in non-supplemented diets, genetic and physiological differences among hens can significantly shape yolk lipid profiles through metabolic modulation [25]. These differences may be influenced by physiological mechanisms related to hepatic lipid metabolism. In laying hens, the liver is the main site for fatty acid synthesis destined for yolk deposition. Although no molecular analyses were performed in this study, variations in the activity of key enzymes such as stearoyl-CoA desaturase (SCD1), Δ6-, and Δ5-desaturases may potentially contribute to the observed differences in MUFA and EPA levels. This interpretation remains hypothetical and would require targeted molecular validation in future studies [17,26]. The peroxidation index (PI), which estimates lipid susceptibility to oxidative degradation, showed no significant differences among breeds. This suggests effective endogenous antioxidant defenses, potentially supported by tocopherols, carotenoids, and selenium-dependent enzymes [27]. Although no direct data were collected on vegetation type or antioxidant intake, the semi-extensive rearing system may have favored exposure to natural antioxidant sources (e.g., spontaneous vegetation), which—according to previous studies—can contribute to redox balance and lipid protection in eggs. However, this hypothesis remains speculative and should be confirmed by further targeted analyses [27,28].

Although management conditions and cereal-based diets were similar across farms, genotype × environment interactions likely influenced the observed differences [29,30,31,32]. This could reflect potential differences in feed conversion efficiency, although no direct measurements of feed intake or FCR were conducted in this study. Future research should address these aspects to clarify their contribution.

Access to spontaneous vegetation—such as legumes, brassicaceous plants, and aromatic herbs—could have increased the levels of beneficial fatty acids and antioxidants [33,34]. This may reflect a terroir effect—a theoretical concept describing the potential influence of local environmental conditions and genotype on product quality—although no specific environmental or botanical profiling was conducted in this study [5].

The lipid profiles and nutritional indices of the native Sicilian breeds were compared with values reported for commercial hybrids to contextualize their functional potential and highlight their role in sustainable poultry systems.

Cornuta eggs exhibited a particularly favorable lipid profile, especially regarding MUFA content (56.22%) and the n-6/n-3 ratio (7.35), both superior compared to commercial hybrid eggs (Hy-Line and Warren), where average MUFA values range between 40% and 49%, and n-6/n-3 ratios are around 12–16, depending on the diet [35]. The other native Sicilian breeds analyzed, Valplatani and Siciliana, although showing a lower MUFA content (48.16% for both), presented values comparable to or competitive with those of commercial eggs.

These findings gain further relevance when placed in the context of commercial hybrid layers commonly used in industrial egg production. For instance, Attia et al. [36] and González-Muñoz et al. [35] reported atherogenic index (AI) values between 1.0 and 1.5 and thrombogenic index (TI) values ranging from 2.0 to 3.0 in Hy-Line and Warren hens—values that are markedly higher than those observed in Cornuta eggs (AI: 0.52; TI: 1.22), highlighting the superior lipid quality of this native breed. In Isa Brown hens, Dedousi et al. [37,38] demonstrated that dietary supplementation with dried olive pulp improved yolk fatty acid profiles and reduced the n-6/n-3 ratio, confirming that nutritional interventions can modulate lipid composition even in highly selected genotypes. Additionally, Rodríguez-Hernández et al. [39] recently analyzed Hy-Line Brown hens reared under both conventional cage and cage-free systems and reported mono-unsaturated fatty acid (MUFA) contents of approximately 44% and PUFA contents of 20–21%, with n-6/n-3 ratios ranging from 11 to 13—values similar to or less favorable than those observed in Cornuta eggs. These comparisons support the idea that native breeds may offer superior nutritional quality and reinforce their role in sustainable and functional food systems.

**Figure 4:**
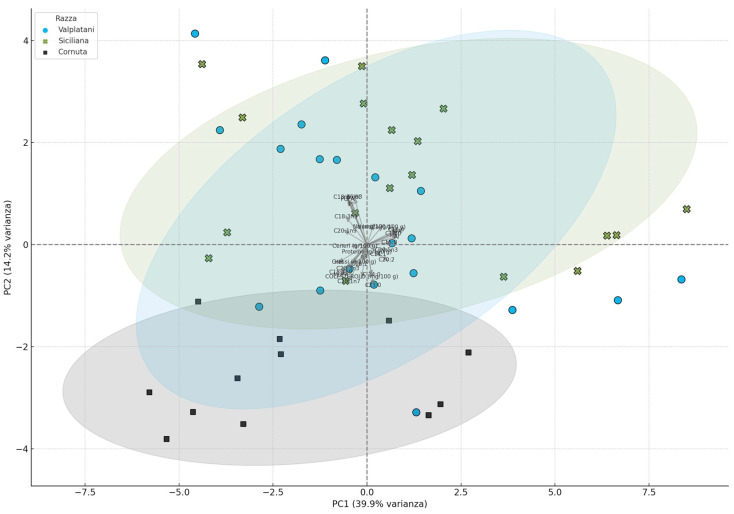
Principal component analysis (PCA) based on eggs chemical and nutritional composition. PCA results were visualized using biplots showing sample distribution by breed and the contribution of each variable (loadings). Ellipses were used to visualize group clustering.

To provide an integrated view of the complex relationships between chemical and nutritional parameters, a principal component analysis (PCA) was conducted. The resulting biplot (Figure 4) showed a clear separation among the three breeds, with Cornuta forming a distinct cluster driven by MUFA, AI, TI, and sodium vectors. The ellipses used to represent the confidence intervals of each group further confirmed the clustering tendency. Variable loadings helped to identify the key contributors to the separation, reinforcing the discriminative value of lipid composition and sodium content. The multivariate structure highlighted in the PCA complements the univariate findings and supports the functional potential of Cornuta eggs. This visualization strengthens the hypothesis that breed-specific metabolic mechanisms influence nutrient deposition and lipid biosynthesis.

Within a framework of moderate and regular consumption—e.g., one egg per day—Cornuta eggs may serve as valuable dietary sources of omega-3 fatty acids and MUFA. Their favorable lipid profile, along with genetic and environmental specificity, supports their potential positioning as functional foods.

As consumers increasingly favor natural, local, and health-promoting products, these eggs could be effectively positioned through voluntary labeling schemes such as “biodiversity-sourced” or “functional egg” [40].

Supporting the conservation and production of these breeds aligns with broader objectives in sustainability, public health, and agricultural diversification. Cornuta represents a model of how natural selection, environmental adaptation, and low-input systems could yield animal products with a favorable lipid profile.

Integration into niche food chains—supported by origin-based claims and nutritional valorization—may thus represent a sustainable strategy for promoting Sicilian poultry biodiversity. This approach complements contemporary goals in agroecology, nutrition, and genetic conservation [41].

## 4. Conclusions

This study provides the first in-depth nutritional characterization of eggs from three Sicilian native chicken breeds reared under semi-extensive conditions. In particular, Cornuta eggs exhibited a more favorable lipid profile, with higher levels of monounsaturated fatty acids, a more balanced omega-6/omega-3 ratio, and improved nutritional indices (AI, TI, HH). Although environmental and dietary conditions were similar across farms, the observed differences suggest a likely genetic influence on egg composition.

These findings contribute to the nutritional valorization of native poultry breeds, highlighting their potential integration into food chains focused on quality and sustainability. The lipid traits observed—especially in Cornuta—are consistent with a nutritional profile generally considered more favorable for health. However, assigning a strictly functional role would require further evidence on physiological effects in humans. In this context, the eggs of the studied breeds may be regarded as traditional products with high nutritional quality, potentially suitable for market differentiation strategies, particularly in local and short supply chains.

Finally, the data generated in this study may serve as a scientific basis for policy actions supporting the inclusion of native breeds in quality schemes, agri-environmental payments, and dietary guidelines. The integration of nutritional quality, genetic conservation, and territorial sustainability is consistent with the FAO Global Plan of Action for Animal Genetic Resources [42], the European Union’s Farm to Fork Strategy [43], and the United Nations 2030 Agenda for Sustainable Development [41].

## Figures and Tables

**Table 1:** Chemical and nutritional composition of eggs from three Sicilian chicken breeds.

Parameter	Siciliana (Mean ± SD)	Cornuta (Mean ± SD)	Valplatani (Mean ± SD)	*p* -Value
Samples	61	59	60	-
Energy (kJ/100 g)	545–131	616–148	596–143	ns
Moisture (%)	75.43 ^a^ ± 1.85	72.86 ^b^ ± 2.32	73.66 ^ab^ ± 1.77	<0.05
Fat (%)	9.08 ^a^ ± 1.55	10.95 ^b^ ± 1.26	10.33 ^ab^ ± 0.68	<0.05
Protein (%)	12.29 ± 0.87	12.40 ± 0.96	12.57 ± 0.63	ns
Ash (%)	1.29 ± 0.29	1.40 ± 0.31	1.46 ± 0.23	ns
Cholesterol (mg/100 g yolk)	1464 ^ac^ ± 52	1789 ^b^ ± 48	1477 ^ac^ ± 50	<0.05
Salt (%)	0.33 ± 0.02	0.33 ± 0.01	0.35 ± 0.02	ns

Different superscript letters (^a–c^) within a row indicate statistically significant differences among breeds (*p* < 0.05). Values are expressed as g/100 g of edible portion (mean ± standard deviation). The Cholesterol value is expressed as mg per 100 g of yolk. ns = not significant.

**Table 2:** Mineral composition of three Sicilian chicken breeds.

Element	Siciliana (Mean ± SD)	Cornuta (Mean ± SD)	Valplatani (Mean ± SD)	*p*-Value
**Samples**	**61**	**59**	**60**	-
Sodium	133.54 ^a^ ± 9.72	133.20 ^a^ ± 5.69	139.92 ^b^ ± 6.51	<0.05
Potassium	142.60 ± 5.34	141.33 ± 10.52	143.98 ± 8.92	ns
Phosphorus	216.10 ± 34.42	219.36 ± 12.87	224.24 ± 17.68	ns
Calcium	58.03 ± 11.79	58.35 ± 6.84	59.44 ± 5.41	ns
Magnesium	12.55 ± 1.78	12.58 ± 1.00	12.34 ± 0.87	ns
Iron	2.55 ± 1.14	2.63 ± 0.58	2.19 ± 0.33	ns
Zinc	1.23 ± 0.24	1.27 ± 0.22	1.26 ± 0.21	ns
Copper	0.16 ± 0.08	0.08 ± 0.01	0.10 ± 0.06	ns
Selenium	0.01 ± 0.00	0.01 ± 0.00	0.01 ± 0.00	ns
Manganese	0.04 ± 0.01	0.04 ± 0.01	0.04 ± 0.01	ns

Different superscript letters (^a,b^) within a row indicate statistically significant differences among breeds (*p* < 0.05). Mineral content is expressed as mg/100 g of edible portion. ns = not significant.

**Table 3:** Fatty acid profile and acid classes (%) of yolk from three Sicilian chicken breeds.

Fatty Acid(%)	Siciliana (Mean ± SD)	Cornuta (Mean ± SD)	Valplatani (Mean ± SD)	*p*-Value
**Samples**	**61**	**59**	**60**	**-**
C14:0	1.10 ^a^ ± 0.09	0.95 ^b^ ± 0.08	1.02 ^a^ ± 0.09	<0.05
C16:0	37.19 ^a^ ± 1.8	28.31 ^b^ ± 1.5	34.79 ^a^ ± 1.7	<0.05
C18:0	10.52 ± 0.8	9.29 ± 0.7	9.88 ± 0.8	ns
C18:1 n-9	39.92 ^a^ ± 2.1	50.74 ^b^ ± 1.9	43.36 ^a^ ± 2.0	<0.05
C18:2 n-6	8.40 ^a^ ± 0.7	6.58 ^b^ ± 0.6	7.95 ^a^ ± 0.7	<0.05
C18:3 n-3	0.63 ^a^ ± 0.05	0.90 ^b^ ± 0.06	0.68 ^a^ ± 0.05	<0.05
C20:4 n-6	1.45 ± 0.12	1.21 ± 0.10	1.34 ± 0.11	ns
C20:5 n-3	0.21 ^a^ ± 0.02	0.41 ^b^ ± 0.03	0.31 ^a^ ± 0.02	<0.05
SFA	48.81 ^a^ ± 8.24	38.55 ^b^ ± 6.93	45.92 ^a^ ± 7.15	<0.05
MUFA	48.16 ^a^ ± 5.91	56.22 ^b^ ± 5.49	48.16 ^a^ ± 5.91	<0.05
PUFA	6.40 ± 2.62	5.03 ± 2.27	5.84 ± 3.10	ns
Omega 3	6.18 ± 2.71	4.45 ± 2.18	5.32 ± 3.16	ns
Omega 6	0.49 ± 0.24	0.61 ± 0.22	0.54 ± 0.28	ns

Different superscript letters (^a,b^) within a row indicate statistically significant differences among breeds (*p* < 0.05). Fatty acid values are expressed as a percentage of total fatty acids (% of total FAMEs). SFA = saturated fatty acids; MUFA = monounsaturated fatty acids; PUFA = polyunsaturated fatty acids. ns = not significant.

**Table 4:** Nutritional indices of yolk from three Sicilian chicken breeds.

Index	Siciliana	Cornuta	Valplatani	*p*-Value
n-6/n-3	13.21 ^a^	7.35 ^b^	12.34 ^a^	<0.05
AI	0.82 ^a^	0.52 ^b^	0.72 ^a^	<0.05
TI	1.91 ^a^	1.22 ^b^	1.66 ^a^	<0.05
HH	1.30 ^a^	2.02 ^b^	1.44 ^a^	<0.05
PI	25.1	24.8	25.0	ns

Different superscript letters (^a,b^) within a row indicate statistically significant differences among breeds (*p* < 0.05). AI = atherogenic index; TI = thrombogenic index; HH = hypocholesterolemic/hypercholesterolemic ratio; n-6/n-3 = omega-6/omega-3 fatty acid ratio. ns = not significant.

## Data Availability

The original contributions presented in the study are included in the article, further inquiries can be directed to the corresponding author.
